# Supplementary material for: Integrated treatment of hepatitis C virus infection among people who inject drugs: A multicenter randomized controlled trial (INTRO-HCV)
Source: PLoS Med. 2021 Jun 1;18(6):e1003653. doi: 10.1371/journal.pmed.1003653 (PMC8205181; doi:10.1371/journal.pmed.1003653)
Supplement: S1 Text — HCV, hepatitis C virus. (PDF) [file pmed.1003653.s001.pdf]

Supporting information file for *Integrated Treatment of Hepatitis C Virus Infection Among People Who Inject Drugs: A Multi-Center Randomized Controlled Trial (INTRO-HCV)*

S1 Text: Norwegian national HCV treatment guidelines during the study period:

Several guideline documents on HCV treatment in Norway have been relevant in the period 2017–2019. The following sources are some of the key documents in choice of treatment (most are in Norwegian and some of these have restricted access):

Guidelines from the Norwegian association for infectious diseases, version 7 (2017): https://www.hepatittfag.no/pdf-hcv

Version 8 (2019): https://www.legeforeningen.no/contentassets/7bdf07c45b95411aa3c66e27822da583/veileder-revisjon-8-1.pdf

The treatment choice guidelines from the health authorities:
Period 01.03.2016–28.02.2017: http://innsiden.helse-bergen.no/komiteer/legemiddelkomiteen/_layouts/WopiFrame.aspx?sourcedoc=/komiteer/legemiddelkomiteen/Dokumentbibliotek%20lager/Informasjon%20om%20LIS-anbud/2016/LIS-anbefalinger%20Hepatitt%20C%202016%20-%20HBe.docx&action=default&Source=http%3A%2F%2Finnsiden%2Ehelse%2Dbergen%2Eno%2Fkomiteer%2Flegemiddelkomiteen%2FDokumentbibliotek%2520lager%2FForms%2FAllItems%2Easpx%3FRootFolder%3D%252Fkomiteer%252Flegemiddelkomiteen%252FDokumentbibliotek%2520lager%252FInformasjon%2520om%2520LIS%252Danbud%252F2016%26FolderCTID%3D0x01200087294102752312459lCBCF04418E246C%26View%3D%7B4E86623D%2D3B87%2D484B%2DA087%2D07D7BB213411%7D&DefaultItemOpen=1

Period 01.03.2017–28.02.2018: http://innsiden.helse-bergen.no/komiteer/legemiddelkomiteen/_layouts/WopiFrame.aspx?sourcedoc=/komiteer/legemiddelkomiteen/Dokumentbibliotek%20lager/Helseforetakenes%20LIS%20HCV%20anbefalinger%202017%20-%20reviderte%20130617.pdf&action=default&Source=http%3A%2F%2Finnsiden%2Ehelse%2Dbergen%2Eno%2Fkomiteer%2Flegemiddelkomiteen%2FDokumentbibliotek%2520lager%2FForms%2FAllItems%2Easpx&DefaultItemOpen=1

Period 01.02.2018–31.01.2019: http://innsiden.helse-bergen.no/komiteer/legemiddelkomiteen/_layouts/WopiFrame2.aspx?sourcedoc=/komiteer/legemiddelkomiteen/Dokumentbibliotek%20lager/Informasjon%20om%20LIS-anbud/2018/LIS%20HCV%20anbefalinger%202018.pdf&action=default&Source=http%3A%2F%2Finnsiden%2Ehelse%2Dbergen%2Eno%2Fkomiteer%2Flegemiddelkomiteen%2FDokumentbibliotek%2520lager%2FForms%2FAllItems%2Easpx%3FRootFolder%3D%252Fkomiteer%252Flegemiddelkomiteen%252FDokumentbibliotek%2520lager%252FInformasjon%2520om%2520LIS%252Danbud%252F2018%26FolderCTID%3D0x01200087294102752312459lCBCF04418E246C%26View%3D%7B4E86623D%2D3B87%2D484B%2DA087%2D07D7BB213411%7D&DefaultItemOpen=1&DefaultItemOpen=1

Period 01.02.2019–31.01.2021: https://sykehusinnkjop.no/seksjon/avtaler-legemidler/Documents/Hepatitt%20B%20og%20C/Anbefaling%20LIS%201908%20hepatitt%20C.pdf

Supporting information file for *Integrated Treatment of Hepatitis C Virus Infection Among People Who Inject Drugs: A Multi-Center Randomized Controlled Trial (INTRO-HCV)*

Data sharing: Description of shared data file.

A comma separated variable file (*HCV-primary-analyses-export-data-sharing-v2.csv*) including the following variables is available:

- HCV PCR 12 weeks after treatment with valid measures (*hcvrna*)
    - 0 (negative)/1 (positive)
- HCV PCR 12 weeks after treatment with assumptions when non-valid (*hcvrnamisno*)
    - 0 (negative)/1 (positive)
- Arm (*arm*, standard [0], integrated [1])
- Gender (*gender*, female/male)
- Treatment initation (*starttx*, no [0], yes [1])
- Treatment initation within 1 year (*starttx1y*, no [0], yes [1])
- Medication used (*medication*)
- Time to treatment delay variable (*total_delay1*)
- Cirrhosis (*cirrhosis*, no [0], yes [1])
- Fibrosis not cirrhosis (*fibrosisnotcirr*, no [0], yes [1])
- Protocol violations (*perprotocoldrop*)

Some variables including age, unstable living conditions, substance use patterns, work/social security, opioid agonist therapy, body mass index, education and date variables for calculating time-to-treatment-initiation are removed, as these might have made it possible to identify respondents.
