# Supplementary material for: Integrated treatment of hepatitis C virus infection among people who inject drugs: A multicenter randomized controlled trial (INTRO-HCV)
Source: PLoS Med. 2021 Jun 1;18(6):e1003653. doi: 10.1371/journal.pmed.1003653 (PMC8205181; doi:10.1371/journal.pmed.1003653)
Supplement: S1 Table — ITT, intention-to-treat; PP, per protocol; SRp, Schoenfeld residuals p-values. (PDF) [file pmed.1003653.s002.pdf]

Supporting information file for *Integrated Treatment of Hepatitis C Virus Infection Among People Who Inject Drugs: A Multi-Center Randomized Controlled Trial (INTRO-HCV)*

S1 Table: Testing of proportional-hazards assumption in Cox regression for effect of integrated vs standard treatment with Schoenfeld residuals p-values (SRp) and sensitivity analyses with split time for treatment initiation with intention-to-treat (ITT) and per protocol (PP) analyses.

ITT	Time 0-2m	1.4 (1.0-2.1, SRp=0.6583)
ITT	Time 2-6m	3.5 (2.4-5.1, SRp=0.3230)
ITT	Time 6-12m	2.2 (1.0-4.8, SRp=0.2039)
ITT	**Overall**	**2.2 (1.7-2.9, SRp=0.0681)**
PP	Time 0-2m	1.2 (0.8-1.8, SRp=0.6037)
PP	Time 2-6m	3.0 (2.0-4.4, SRp=0.2231)
PP	Time 6-12m	2.3 (1.0-5.2, SRp=0.3231)
**PP**	**Overall**	**1.9 (1.5-2.5, SRp=0.0433)**
