# Supplementary material for: Integrated treatment of hepatitis C virus infection among people who inject drugs: A multicenter randomized controlled trial (INTRO-HCV)
Source: PLoS Med. 2021 Jun 1;18(6):e1003653. doi: 10.1371/journal.pmed.1003653 (PMC8205181; doi:10.1371/journal.pmed.1003653)
Supplement: S2 Fig — Subgroup effects are presented for gender, the type of treatment center, age group, living condition, injecting drug use behavior the last 6 months, fibrosis and cirrhosis, and overall effects (without and with cluster adjustment). PP, per protocol; SVR, sustained virologic response. (PDF) [file pmed.1003653.s004.pdf]

Supporting information file for *Integrated Treatment of Hepatitis C Virus Infection Among People Who Inject Drugs: A Multi-Center Randomized Controlled Trial (INTRO-HCV)*

S2 Fig: Sub-group analyses for binomial logit regression of sustained virologic response of hepatitis C for those who initiated treatment (post hoc per protocol analyses). Sub-group effects are presented for gender, the type of treatment center, age group, living condition, injecting drug use behavior the last 6 months, fibrosis and cirrhosis, and overall effects (without and with cluster adjustment).

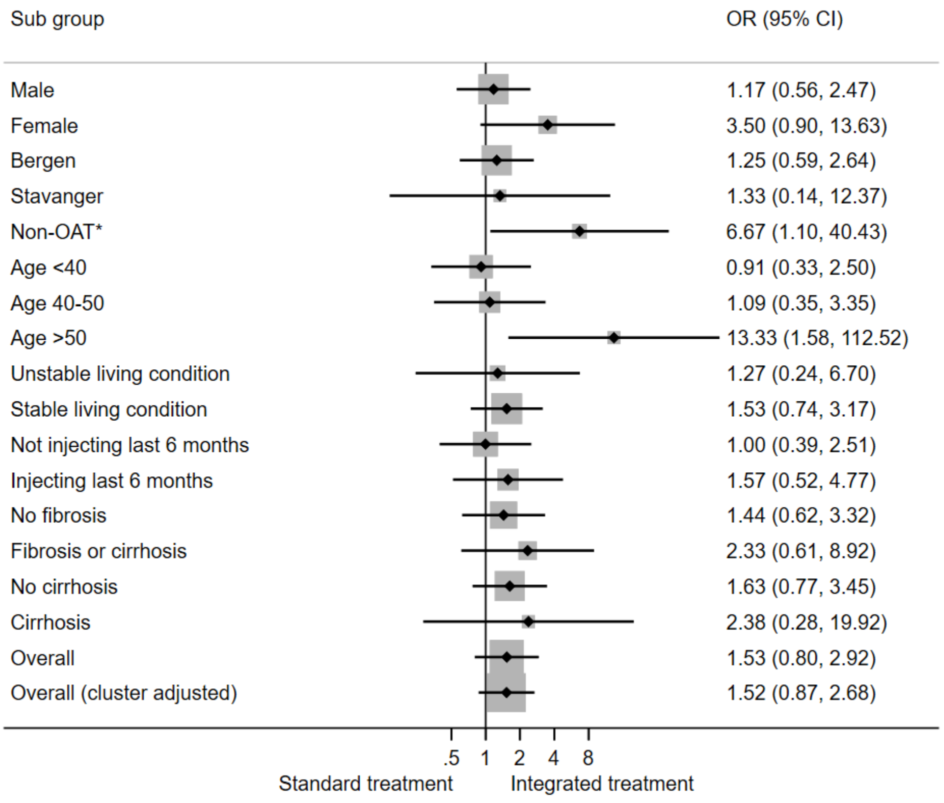

* Non-OAT: patients who did not receive opioid agonist therapy
