# Supplementary material for: Integrated treatment of hepatitis C virus infection among people who inject drugs: A multicenter randomized controlled trial (INTRO-HCV)
Source: PLoS Med. 2021 Jun 1;18(6):e1003653. doi: 10.1371/journal.pmed.1003653 (PMC8205181; doi:10.1371/journal.pmed.1003653)

Supporting information file for *Integrated Treatment of Hepatitis C Virus Infection Among People Who Inject Drugs: A Multi-Center Randomized Controlled Trial (INTRO-HCV)*

S4 Fig: Time to hepatitis C treatment initiation for genotype 3. Red line/area indicates proportion of participants in integrated arm initiation treatment with confidence intervals. Blue dashed line/area indicates proportion of participants in treatment as usual arm initiation treatment.

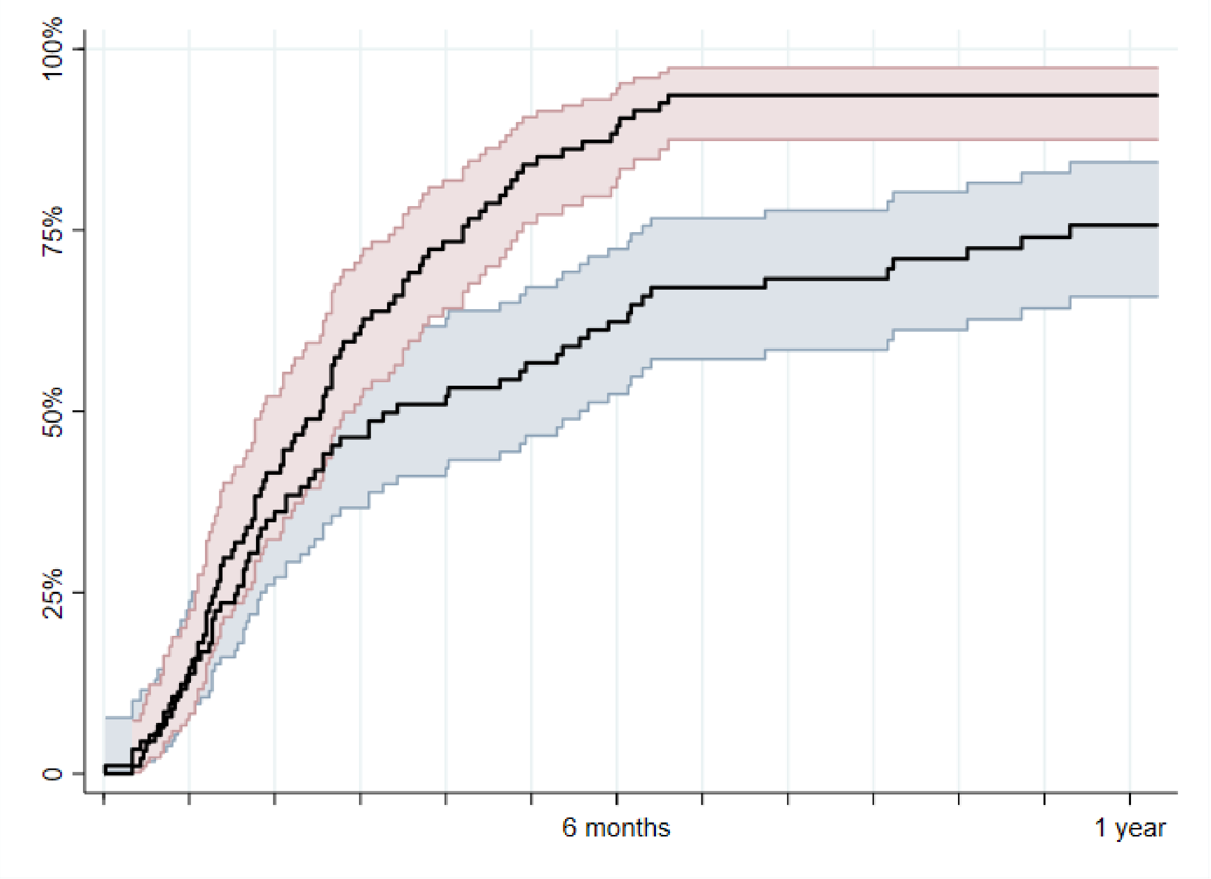

Supplement: S4 Fig — (PDF) [file pmed.1003653.s006.pdf]
