# Supplementary material for: Integrated treatment of hepatitis C virus infection among people who inject drugs: A multicenter randomized controlled trial (INTRO-HCV)
Source: PLoS Med. 2021 Jun 1;18(6):e1003653. doi: 10.1371/journal.pmed.1003653 (PMC8205181; doi:10.1371/journal.pmed.1003653)
Supplement: Attachment — Submitted filename: Response-INTRO-HCV-LTF-rev-3.pdf [file pmed.1003653.s009.pdf]

Haukeland University Hospital

Bergen Addiction Research, Department of Addiction Medicine, Haukeland University Hospital

Comments from the reviewer 3:

Dear Reviewer,

Thank you for your constructive feedback on our paper, entitled "*Integrated treatment of hepatitis C virus infection among people who inject drugs: a multi-center randomized controlled trial (INTRO-HCV*" under consideration in PLOS Medicine. We have revised our manuscript based on the comments and suggestions from the reviewers and editors and have provided specific responses below (your comments in italicized grey fonts and our responses in black fonts). Two manuscript files are included: A "clean" version in one file, along with a second file where all revisions are displayed with tracked changes.

Reviewer #3: This is a well designed and conceptualised RCT of integrated HCV treatment by multidisciplinary teams in opioid treatment settings, which will make a worthwhile contribution to the literature. The statistical approaches are sound.

Response: Thanks for these comments.

The mention of COVID-19 in the introduction is interesting but requires more in-depth consideration to be meaningful. In particular, the statement "This could even become essential if, for example a vaccine for COVID-19 should be developed with a three dosage regime" requires elaboration so that it is clear why better evidence on models of care is essential in this context. The study findings (discussion section) also need to be considered in the context of COVID-19 if inclusion of these details is going to be meaningful.

Response: We see your point and have now removed the two sentences regarding COVID-19 to avoid confusion.

There are a number of small revisions that should be considered.
1. Methodology: It would help (for understanding study validity) to briefly summarise in the text the details on non-response that are included in Figure 1. How many people were seen at the relevant services (OAT/CCC) during the study recruitment period, what proportion were eligible to participate and of these how many actually participated? Did those who participated differ in any significant way from those who refused or were unavailable? (If you can distinguish between non-availability and ineligibility). There are a reasonable number who declined consent, so it would help to know if they had different characteristics. What proportion overall were lost to follow up?

Response: We have now added some more details from the trial profile in the methods section and a small note in the limitation section. To summarize, The target populations were PWID at OAT clinics in Bergen (n=901), Stavanger (n=130), and community care centers (CCC) in Bergen (n=208). Of these, 136/1237 (11%) did not consent for participation and study assessment. We do not have data on those not consenting to participate. A total of 298 had untreated HCV and no exclusion criteria and were valid for randomization.

Design and limitations:
2. Hospital clinics often differ from specialised (e.g. OAT) and community based clinics in important ways other than the range services provided on-site, e.g. service culture/philosophy and social environment (e.g. stigma). The physical environment may also differ. It was noted in the methods section that people randomised to the hospital outpatient condition had to travel independently to the hospital site. People who inject drugs have sometimes reported that hospital clinics are more difficult to access than community based clinics, partly because these settings seem more formal and daunting. It should be considered in the study limitations that some of the group differences could be explained by the study settings.

Response: Very good points, and we have now added some sentences on this in the discussion to be more explicit about study settings.

3. On a more nuanced level, there is also the question of the intervention elements, i.e. integration and multidisciplinary care. To what extent are the treatment effects attributable to the easy on site access for OAT clients treated at an integrated service, rather than the additional support and engagement offered by a multidisciplinary team? More detail about the composition of the teams (in the context of this study) and the services they provided would be helpful. It may not necessarily be possible to fully address the role of the different intervention elements, but the question should at least be considered.

Response: We have now added some sentences on this in the methods and discussion section.

4. In the discussion it would be worthwhile briefly reflect on the limitations of 12 week SVR as a measure of treatment completion and cure. Rates of SVR testing in PWID are often around 80-85% but are unlikely to represent true outcomes.

Response: We have now added some sentences on this in the discussion.

5. The written expression is generally OK but the paper could use a thorough proof read. There are some errors in grammar and expression, e.g. Abstract: "The aim of the study was to evaluates the efficacy of integrated treatment of HCV infection

among PWID with standard treatment" and "Among 298 included participants people randomized to standard treatment" and "Integrated treatment for HCV in was superior to….." and in the Discussion (p. 9; spelling) "Among those who initiated treatment in our trial, 84% achieved SVR which is slightly fewer compared to a trial on direct acting antiviral medications were 92% achieved SVR" ('were' should be 'where').

Response: We see as you clearly point out that there were language errors. Thanks for noting these. We have now revised the aforementioned sentences and two native English speakers have also carefully proof read the revised manuscript.

6. Page 5: "…eligible for treatment according to national HCV treatment guidelines". Is it possible to provide a reference to these guidelines (or append)?

Response: We have now added references for these guidelines.

7. Randomization and masking: Please briefly specify the masking measures within this paper; this an important detail and the reader shouldn't need to refer to another paper for this.

Response: We have now added some more details on this. To summarize, randomization was disclosed to clinical staff providing treatment and follow-up for PWID. Patients were informed about key elements in the delivery of the respective intervention and follow-up that they were randomized to, but no information was provided about the treatment and follow-up alternatives in the other arm, nor what the hypotheses for the study were.

8. You should consider noting the treatment side effects in the discussion because these have implications for client education, engagement, and treatment management.

Response: We have now added a sentence on this in the discussion.

Andrew Smirnov, The University of Queensland.
