# Supplementary material for: Integrated treatment of hepatitis C virus infection among people who inject drugs: A multicenter randomized controlled trial (INTRO-HCV)
Source: PLoS Med. 2021 Jun 1;18(6):e1003653. doi: 10.1371/journal.pmed.1003653 (PMC8205181; doi:10.1371/journal.pmed.1003653)
Supplement: Attachment — Submitted filename: Response-INTRO-HCV-LTF-2021-03-R2.pdf [file pmed.1003653.s010.pdf]

Haukeland University Hospital

Bergen Addiction Research, Department of Addiction Medicine, Haukeland University Hospital

Comments from the reviewer 2: *Alex McConnachie, Statistical Review*

Dear Reviewer,
Thank you for your constructive feedback on our paper, entitled "*Integrated treatment of hepatitis C virus infection among people who inject drugs: a multi-center randomized controlled trial (INTRO-HCV*" under consideration in PLOS Medicine. We have revised our manuscript based on your comments and suggestions from the reviewers and editors and have provided specific responses below (your comments in italicized grey fonts and our responses in black fonts). Two manuscript files are included: A "clean" version in one file, along with a second file where all revisions are displayed with tracked changes.

I thank the authors for their consideration of my original comments. I am happy with almost all of their responses. The only thing that I feel is still lacking are interaction tests for the subgroup analyses presented in Figure 3 and 4. All that would be required is a column of interaction p-values down the right hand side of each figure. Less importantly, each group of estimates and CIs could be separated slightly from the others - this would make it easier to follow

Response: Thanks for these comments. We have now presented interaction tests for the subgroup analyses presented in text below Figure 3 and 4.

Regarding increasing space between each group of categories in the figures, we are not aware if this can be done directly in the Stata-based figure we have made. However, if it is possible for the journal to process the image files using the vector files we provided to increase spacing between the different categories, that would be fine for us. If this is difficult, we think the figures should be possible to follow them as they are.
